# Mineral Products of the United States for the Years 1886 to 1895

**Published:** 1896-09

**Authors:** 


### MINERAL PRODUCTS OF THE UNITED STATES FOR THE CALENDAR YEARS 1886 TO 1895. Department of the Interior, United States Geological Survey, Charles D. Walcott, Director; David T. Day, Chief of Division of Mineral Resources, Washington, D. C., August 1st, 1896.

This publication is a large chart containing tabular reports of the various minerals produced in the United States.

It is divided into two main parts—Metallic and Non-Metallic products.

It shows the quantity and the value of each mineral produced for each of the years in the decade from 1886 to 1895, inclusive. It would be an impossible task to analyze the results given in this table; we can only commend it for its elaborate and clearly presented details, any of which can be found at a glance, owing to its systematic arrangement. For those engaged in mining, for the statesman, engaged in formulating just tariff schedules; for the railroad man, fixing freight rates, and for the political economist, it is an invaluable document.

